# Supplementary material for: Health status of street children and reasons for being forced to live on the streets in Harar, Eastern Ethiopia. Using mixed methods
Source: PLoS One. 2022 Mar 18;17(3):e0265601. doi: 10.1371/journal.pone.0265601 (PMC8932583; doi:10.1371/journal.pone.0265601)
Supplement: S1 Table — (PDF) [file pone.0265601.s001.pdf]

Supplementary file: Table: Distribution of sample interviewees by selected sites in the Harar, eastern Ethiopia, 2021

S. No	Name of the Sub town	Total of registered [illegible]	Number of the interviewees
1	Arategna	50	23
2	Agip	61	28
3	Canal	53	24
4	Bote	92	42
5	Shewa Ber	101	46
6	Andegan Menged	99	44
7	Feres megala	46	21
	Total	502	228
