# Supplementary material for: Health status of street children and reasons for being forced to live on the streets in Harar, Eastern Ethiopia. Using mixed methods
Source: PLoS One. 2022 Mar 18;17(3):e0265601. doi: 10.1371/journal.pone.0265601 (PMC8932583; doi:10.1371/journal.pone.0265601)
Supplement: S1 File — (PDF) [file pone.0265601.s002.pdf]

S1 file- Data collection questionary

Questionnaire code no: ____________

Data collector name: _______________

S. No	Questions	Response	Remark
**Socio-demographic characteristics of study participant**			
**101**	Age (in years)	_____	
**102**	Gender	1. Male<br>2. Female	
**103**	Present address	1. Urban<br>2. Rural	
**104**	Place of birth	_______	
**105**	Ethnicity	1. Oromo<br>2. Amhara<br>3. Harari<br>4. Other, specify/__________/	
**106**	Religion	1. Muslim<br>2. Orthodox<br>3. Protestant<br>4. Other, specify/__________/	
**107**	What is your current marital status?	1. Currently married<br>2. Never married<br>3. Divorced<br>4. Widowed<br>5. Others, specify /_______________/	
**Education background of the child**			

**201**	Educational status	1. Never attend school<br>2. Only read and write<br>3. 1 -4 grade<br>4. 5 – 8 grade<br>5. 9 -12 grade	
**202**	If you have never been enrolled to school, what factors hindered you from attending it?	1. Family poverty/Financial constraints<br>2. No school available nearby<br>3. Family did not attach value to education<br>4. Had to remain home to help the family<br>5. Others (specify)	
**203**	If you are school dropout now, why did you give up schooling?	1. Family could no longer afford school fees<br>2. I had to work to supplement family income<br>3. Poor academic performance<br>4. Health/medical reasons<br>5. Expelled because of conduct<br>6. To make my own reading<br>7. Others (specify)	
**204**	If you are given the chance to continue your education now, are you willing to and happy to make use of the opportunity?	1. Yes<br>2. No	
**Family background of the interviewee**			
**301**	Do you have parents?	1. Yes<br>2. No	
**302**	Are your parents alive?	1. Both alive	

		2. both are dead<br>3. Only mother alive<br>4. Only father alive<br>5. Do not know	
**303**	If both are still alive, what was/is the marital status of your parents?	1. Marries and living together<br>2. Divorced due to bad marriage arrangements<br>3. Circumstantial separation (war, work displacement…)<br>4. Widowed<br>5. Single parent<br>6. Separated because of death<br>7. Don’t know<br>8. Others (specify)	
**304**	If divorced, in your opinion what was the cause of the divorce?	1. Poverty<br>2. father left home to look for a job<br>3. Bad habit<br>4. others (specify)	
**305**	What is/was your mother’s means of livelihood?	1. informal daily worker<br>2. petty seller/trader<br>3. skilled worker/self-employed<br>4. house servant<br>5. beggar<br>6. house wife<br>7. other (specify)	
**306**	What is/was your father’s means of income/livelihood?	1. Farming<br>2. Informal daily labourer<br>3. Trader<br>4. Government employee<br>5. private employee<br>6. beggar	

		7. soldier/x-soldier<br>8. guard<br>9. other (specify)	
**307**	Where do your parents live now?	1. In Harar<br>2. In another urban town<br>3. In rural areas<br>4. Don’t know<br>5. If in Harar, please state residential area, i.e., Woreda, Kebele? (If any)	
**308**	Who supports the family now?	1. Father Only<br>2. Mother only<br>3. both mother & father<br>4. Extended family<br>5. Myself<br>6. Other (specify)	
**309**	If your parents are migrants, why did they leave their place of origin?	1. Drought/famine situation<br>2. To look for a job<br>3. For medical treatment<br>4. Don’t know<br>5. Others (specify)	
**310**	What is your parents’ monthly income? Approximately	1. <1000<br>2. 1000-2000<br>3. >2000<br>4. I don’t know	
**311**	What is the educational status of your father?	1. Illiterate<br>2. Can read and write<br>3. Highest grade completed	
**312**	In what type of a house are your parents living now?	1. Brick stone<br>2. Bamboo sticks<br>3. Mud house	

		4. Plastic/scrap<br>5. Tin<br>6. Other (specify) ______________	
**Present life style/circumstances of the child**			
**401**	For how long have you been engaged in street way of life? (Duration of street life)	1. ____Yrs___ Mos	
**402**	What factors forced you to resort to street way of life?	1. Peer pressure<br>2. To look for a job<br>3. Quarreled with parents<br>4. In search of food<br>5. Forced<br>6. Deaths of parents<br>7. Lack of peace in the family<br>8. Failure of family to support me<br>9. Other (specify)________	
**403**	Do you work?	1. Yes<br>2. No	
**404**	If your answer is no, how do you survive?	____________	
**405**	What is your street occupation?	1. Begging<br>2. Shoe shinning<br>3. Carrying small items<br>4. Delivering messages<br>5. peddler<br>6. Taxi boy<br>7. Other (specify) ______________	
**406**	How much do you get on the average per day?	1. Less than five birr<br>2. 5 -10 birr<br>3. 10 -20 birr	

		4. 30-50 birr<br>5. more than 50 birr	
**407**	How do you spend your daily income?	1. Food<br>2. Shelter<br>3. Cloth<br>4. School fee<br>5. Help family<br>6. Other (specify)____________	
**408**	If you are contributing to the family budget, what is your parents' attitude towards your contribution?	1. Happy<br>2. Unhappy<br>3. Indifferent<br>4. Don't know	
**409**	Is your daily income reliable?	1. Yes<br>2. No	
**410**	Do you beg on the streets?	1. Yes<br>2. No	
**411**	If yes, how often?	1. Always<br>2. Often<br>3. Sometimes<br>4. Rarely<br>5. Never	
**412**	With whom were you living before you resort to street life?	1. With both parents<br>2. With my mother<br>3. With father only<br>4. With close relatives	
**413**	With whom are you living now?	1. With my mother<br>2. with both parents<br>3. With relatives<br>4. With father Alone<br>5. With peers/friends<br>6. Other, specify___________	

**414**	Who helps and protects you while you are on the street?	1. No one<br>2. Siblings<br>3. Peer groups<br>4. Parents<br>5. Close relatives	
**415**	How often do you usually see/visit your families?	1. Everyday<br>2. at least once in a week<br>3. Once in every month<br>4. only few times in a year<br>5. Rarely<br>6. Irregularly<br>7. Never	
**416**	Do you think your parents or guardians approve/disapprove of your living and/or working on the streets?	1. Approve<br>2. Disapprove<br>3. Don't care<br>4. Don't know	
**417**	How many times a day do you eat?	1. Once<br>2. Twice<br>3. Trice	
**418**	What kind of food do you usually eat?	_______	
**419**	Where do you usually go to sleep?	1. Home<br>2. church yards<br>3. mosque yards<br>4. Bus stops<br>5. on verandah<br>6. cheap hotels	
**420**	What are the major problems that you currently encounter in your day-to-day street life?		

**421**	Have you ever been sick while living or working on the streets?	1. Yes<br>2. No	
**422**	If yes to Q421, what kind of illness?		
**423**	Whom do you usually call on when and, as you get sick?		
**424**	Do you have any kind of habit/addiction?	1. Yes<br>2. No	
**425**	If yes to Q424, what kind?		
**426**	Have you ever been beaten or hurt by adults while living/working on streets?	1. Yes<br>2. No	
**427**	Has anyone forced you to do any sexual act?	1. Yes<br>2. No	
**428**	Have you ever had any sexual intercourse with someone for the sake of money?	1. Yes<br>2. No	
**429**	Which of the following is your immediate need?	1. Food<br>2. Cloth<br>3. Shelter<br>4. Education<br>5. Family support<br>6. Cash assistance<br>7. Get reunified<br>8. Other (specify)	
**430**	What do you personally think or believe is the most appropriate intervention measure/s that must be undertaken right now in order to help you improve your way of life?		
**Substance and alcohol Abuse**			

**501**	Do you drink alcoholic beverage like Tela, Tej, Beer, Arekie and the likes?	1. Have never drunk<br>2. I have tried once or twice<br>3. I drink most of the time<br>4. I drink daily	
**502**	What initiates you to use alcohol?	1. Depression<br>2. Peer pressure<br>3. To protect hunger<br>4. To protect fear (sex, steal)<br>5. other, specify /_____/	
**503**	Do you use drugs?	1. Yes<br>2. No	
**504**	If yes, which drug do you use most of the time?	1. Chat chewing<br>2. Ganja/Hashish,<br>3. Cigarette smoking<br>4. Sniff Benzene<br>5. Injectable drug<br>6. Other, specify /____________/	
**505**	What initiates you to use drugs?	5. Depression<br>6. Peer pressure<br>7. To protect hunger<br>8. To protect fear (sex, stell)<br>9. other,specify /_________/	
**Risk sexual and Reproductive health practices**			
**601**	Have you ever had sexual intercourse?	1. Yes<br>2. No	
**602**	If yes to Q601, at what age did you first have sexual intercourse?	_____	
**603**	Why did you decide to have sexual intercourse the first time?	1. I get married<br>2. Fell in love.<br>3. Personal desire.	

		4. Coercion (Rape).<br>5. To get money and other gifts.<br>6. Peer pressure<br>7. Influence of khat /alcohol<br>8. Others, specify /_______/	
**604**	With whom did you make your first sexual intercourse?	1. Husband/wife<br>2. with a steady boy/girl friend<br>3. with a casual boy/girl friend<br>4. with a family member<br>5. with commercial sex worker<br>6. others, specify /_______/	
**605**	Have you had sexual intercourse within the past 1 month?	1. Yes<br>2. No	
**606**	How many sexual partners have you ever had?	1. _____<br>2. NA	
**607**	Have you been raped without the consent of you during the past 12 months?	1. Yes<br>2. No	
**608**	If yes, have you used condom or contraceptives?	1. Yes<br>2. No	
**609**	If no to Q608, why not?	1. It was unplanned<br>2. violator opposing<br>3. Too far to get condom or<br>4. Contraceptives (Not accessible)<br>5. Method was expensive<br>6. I have infrequent sex.<br>7. other, specify /_________/	
**610**	What sexual and reproductive health problems you faced with in last 12 months?	1. unwanted pregnancy<br>2. abortion<br>3. alcohol influenced unsafe sex<br>4. STIs	

		5. Coercion (Rape).<br>6. Coercion (Rape) attempted<br>7. No problem<br>8. other, specify /_______/	
**611**	Have you been pregnant? [FOR FEMALES ONLY]	1. Yes<br>2. No	
**612**	Were your pregnancies wanted?	1. Yes<br>2. No	
**613**	If no, how did you become pregnant?	1. Contraceptive method not available<br>2. Coercion (rape)<br>3. Method failure<br>4. lack of emphasis<br>5. Other, specify /__________/	
**614**	Did you give birth?	1. Yes<br>2. No	
**615**	Have you ever had abortion?	1. Yes<br>2. No	
**616**	If yes to Q615, where did you abort?	1. At public health institution<br>2. At private clinic<br>3. At abortionist's house<br>4. I have induced it myself<br>5. Others, specify….	

Thank you

Uunkaa ragaan ittiin guuramu- Afaan Oromoo

Koodii uunkaa af-gaafii: ____________

Maqaa nama ragaa funaanuu: _______________

T/L	Gaafilee	Deebii	Yaada
**Odeeffannoo hirmataa**			
**101**	Umrii (waggaan)	_____	
**102**	Koornayaa	1. Dhiira<br>2. Dhalaa	
**103**	Teessoo hammaa	1. Magaala<br>2. Baadiyyaa	
**104**	Bakka dhalootaa	_______	
**105**	Sabummaa	1. Oromoo<br>2. Amhaara<br>3. Harari<br>4. Kan biroo, ibsi/__________/	
**106**	Amantii	1. Musliima<br>2. Ortodoksii<br>3. Protestantii<br>4. Kan biraa, ibsi/__________/	
**107**	Haalli gaa’ela kee hamma maalinnii?	1. Hamma kan heerumte/fuudhe<br>2. Kan heerumtee/fuudhee hin beekne<br>3. Kan hiikte/hiike<br>4. Kan abbaan manaa/haati manaa irraa du’e/duute<br>5. Kan biraa, ibsi /_______________/	

**Waa’ee barnoota daa’imaa**			
**201**	Haala barnootaa	1. Barumsa hordofee hin beeku<br>2. Dubbisuu fi barreessuu qofa<br>3. Kutaa 1 -4<br>4. Kutaa 5 – 8<br>5. Kutaa 9 -12	
**202**	Hanga hammaa baruumsa hin hordofne yoo ta’e maaltu si dhorge?	1. Hiyyuma maatii/hanqina maallaqaa<br>2. Manni baruumsaa dhiyeenya irraa dhabamuu<br>3. Maatiin fayidaa barumsaa beekuu dhabuu<br>4. Gargaarsa maatiif jecha manatti hafuu<br>5. Kan biraa, ibsi/_______________	
**203**	Hamma barnoota yoo addaan kutte, maalif addaan kutte?	1. Maatiin kanfaltii mana barnootaa dadhabe<br>2. Galii maatii koo deeggaruuf<br>3. Baruumsatti waanin dadhabaa ta’eef<br>4. Rakkoo fayyaa<br>5. Rakkoo naamusaan hari’ame<br>6. Ofii kootif dubbisuuf<br>7. Kan biraa, ibsi_______________	
**204**	Osoo carraan akka barnoota kee itti fuftu siif kennamee itti fufuuf fedhiifi gammachuu qabdaa?	1. Eeyyee<br>2. Lakki	
**Odeeffannoo maatii hirmaataa**			
**301**	Maatii qabdaa?	1. Eeyyee	

		2. Lakki	
**302**	Maatiin kee lubbuun jiruu?	1. Lachuu jiru<br>2. Lachuu du'an<br>3. Haadha qofatu jira<br>4. Abbaa qofatu jira<br>5. Hin beeku	
**303**	Lachuu kan jiran yoo ta'e, haalli gaa'ila isaanii maali irra ture/jiraa?	1. Waliin jiru<br>2. Waliigaltee dhabaan wal hiikanii jiru<br>3. Yeroof adda bahanii jiru (lolaan, hojiin…)<br>4. Irraa du'ee/duutee jira/jirti<br>5. Kophaa<br>6. Du'aan adda bahan<br>7. Hin beeku<br>8. Kan biraa ibsi____________	
**304**	Walhiikanii yoo jiran sababa maalitiin walhiikani?	1. hiyyuummaa<br>2. abbaan hojii barbaacha deeme<br>3. amala badaa<br>4. Kan biraa ibsi____________	
**305**	Galiin haadha keetii maalii?	1. Hojii guyyuu<br>2. Daldaltuu waantota xixiqqoo<br>3. Hojii dhuunfaa<br>4. Hojjaattu mana namaa<br>5. Kadhattuu<br>6. Haadha manaa<br>7. Kan biraa ibsi____________	
**306**	What is/was your father's means of income/livelihood?	1. Qonna<br>2. Dafqaan bulaa<br>3. Daldalaa<br>4. Hojjataa mootummaa	

		5. Hojjataa dhuunfaa<br>6. Kadhataa<br>7. Raayyaa/raayya durii<br>8. Waardiyyaa<br>9. Kan biraa ibsi____________	
**307**	Maatiin kee hamma eessa jiraatuu?	1. Harar keessa<br>2. Magaalaa biro<br>3. Baadiyyaa biroo<br>4. Hin beeku<br>5. Harar keessa yoo tae bakka isaa ibsi, Aanaa____ganda____	
**308**	Maatii hamma eenyutu gargaaraa jiraa?	1. Abbaa koo qofa<br>2. Haadha koo qofa<br>3. Lachuu<br>4. Ana<br>5. Kan biraa ibsi____________	
**309**	Maatiin kee baqattoota yoo ta'an maalif bakka dhalootatii deemanii?	1. Gogiinsaf/beelaf<br>2. Hojii barbaachaf<br>3. Wal'aansa fayyaaf<br>4. Hin beeku<br>5. Kan biro	
**310**	Galiin maatii keetii ji'aan hammamii? tilmaaman	1. <1000<br>2. 1000-2000<br>3. >2000<br>4. Hin beeku	
**311**	Sadarkaan baruumsa abbaa keetii maalinnii ?	4. Baruumsa hin qabu<br>5. Dubbisuuf barreessuu<br>6. Sadarkaa olaanaa kan xumure	
**312**	Maatiin kee mana akkamii keessa jiraachaa jiruu?	1. Mana dhagaa<br>2. Mana muka shimalaa<br>3. Mana dhoqqee	

		4. Pilaastika keessa<br>5. qorqooroo<br>6. Kan biraa ibsi____________	
**Haala jireenya daa'imaa kan yeroo hammaa**			
**401**	Yeroo hammamiif daandii irra jiraattee? (turmaata jireenya daandii)	1. waggaa________fi Ji'a_______<br>2. hin beeku/yaadadhu	
**402**	Sababoota maalitu akka daandiitti baatu si godhe?	1. Dhiibbaa hiriyootaan<br>2. Hojii barbaacha<br>3. Lola maatii waliinii<br>4. Nyaata barbaacha<br>5. Dirqamaan<br>6. Du'a maatii<br>7. Maatii keessatti nageenyi dhabamuu<br>8. Gargaarsa maatii dhabuu<br>9. Kan biraa ibsi____________	
**403**	Hojjattaa?	1. Eeyyee<br>2. Lakki	
**404**	Lakkii yoo ta'e akkamiin jiraattaa?	___________	
**405**	Hojiin kee daandii irraa maalinnii?	1. Kadhaa<br>2. Kophee haxaawuu<br>3. Meeshalee xixiqoo baachuu<br>4. Ergaa ergamuu<br>5. Hanna<br>6. Taaxii<br>7. Kan biraa ibsi____________	
**406**	Guyyaatti maallaqa hammam argattaa?	1. Qarshii 5 gadi<br>2. Qarshii 5 -10<br>3. Qarshii 10 -20<br>4. Qarshii 30-50	

		5. Qarshii 50 ol	
**407**	Galii guyyaa maal fa’aa irra oolchitaa?	1. Nyaata<br>2. Bakka bultii<br>3. Uffata<br>4. Kanfaltii mana barumsaa<br>5. Gargaarsa maatii<br>6. Kan biraa ibsi____________	
**408**	Maatiikee kan gargaartu yoo ta’e, ilaalchi maatii keetii akkamii?	1. Gammadoo dha<br>2. Gammadoo miti<br>3. Adda adda<br>4. Hin beeku	
**409**	Galiin kee guyyaa gahaa dha?	1. Eeyyee<br>2. Lakki	
**410**	Daandii irratti kadhattaa?	1. Eeyyee<br>2. Lakki	
**411**	Eeyyee yoo ta’e hammamiif?	1. Yeroo hunda<br>2. Yeroo baay’ee<br>3. Al tokko tokko<br>4. Darbee darbee<br>5. Gonkumaa	
**412**	Osoo gara daandii hin bahin eenyu waliin jiraachaa turte?	1. Maatii koo lachuu waliin<br>2. Haadha koo waliin<br>3. Abbaa koo waliin<br>4. Fira waliin	
**413**	Yeroo hammaa eenyu waliin jiraattaa?	1. Haadha koo waliin<br>2. Maatii koo lachuu waliin<br>3. Fira waliin<br>4. Abbaa koo waliin<br>5. Hiriyoota waliin<br>6. Kan biraa, ibsi_______	

**414**	Yeroo daandii irra jiraattu eenyutu si gargaara/eegaa?	1. Homtuu<br>2. Ijoollee koo<br>3. Hiriyoota<br>4. Maatii<br>5. Fira dhiyoo	
**415**	Maatii hammamiin ilaaltaa?	1. Guyyaa guyyaan<br>2. Torbeetti altokko<br>3. Ji'atti altokko<br>4. Waggaatti yeroo muraasaf<br>5. Darbee darbee<br>6. Yeroo qabamaan ala<br>7. Gnkumaa	
**416**	Maatiin kee ykn warri si guddisu hojii fi jireenya daandii irraa murteessuu danda'u jettee yaaddaa?	1. Mirkanneessan<br>2. Hin mirkaneessine<br>3. Dhimma isaanii miti<br>4. Ani hin beeku	
**417**	Guyyaatti al meeqa nyaattaa?	4. Al tokko<br>5. Al lama<br>6. Al sadii	
**418**	Nyaata akkamiiti yeroo baay'ee kan nyaattu?	______	
**419**	Irribaaf eessa dhaqxaa?	1. Mana<br>2. Waldaa kiristaanaa<br>3. masqiida<br>4. buufata konkolaataa<br>5. barandaa<br>6. hoteela gatii xiqqaa	
**420**	Rakkooleen gurguddoon jireenya daandii irraatti yeroo hammaa si mudatan maal fa'ii?		

**421**	Yeroo daandii irrra hojjaattu/jiraattu dhukkubsattee beektaa?	1. Eeyyee<br>2. Lakki	
**422**	Deebiin kee Q421 eeyyee yoo ta'e, dhukkuba akkamii?		
**423**	Yoo dhukkubsatte eenyu waamtaa?		
**424**	Araada qabdaa?	1. Eeyyee<br>2. Lakki	
**425**	Deebiin kee gaafii Q424, gosa kamii?		
**426**	Yeroo jireenya daandii nama gurguddaan rukutamtee ykn miidhamtee beektaa?	1. Eeyyee<br>2. Lakki	
**427**	Namni qunnamtii saalaaf si dirqamsiise jiraa?	1. Eeyyee<br>2. Lakki	
**428**	Maallaqaaf jettee qunnamtii saalaa nama waliin gootee beektaa?	1. Eeyyee<br>2. Lakki	
**429**	Yeroo hammaatti atattamaan gargaarsa maal barbaaddaa?	1. Nyaata<br>2. Uffata<br>3. Bakka jireenyaa<br>4. Barumsa<br>5. Gargaarsa maatii<br>6. Gargaarsa maallaqaa<br>7. Maatiin walitti dabalamuu<br>8. Kan biraa, ibsi____________	
**430**	Yeroo hammaatti tarkaanfiin jireenya kee fooyyeessuuf fudhatamuu qaba jettee yaaddu maalinnii		
**Araada adda adda fi fayyadama qoricha seera malee**			
**501**	Dhugaatiiwwan alkoolii akka farsoo, daadhii, biiraa, araqee, fi kkf dhugdaa?	1. Dhugee hin beeku<br>2. Al tokko ykn lama yaalera<br>3. Yeroo baay'ee nan dhuga	

		4. Guyyaanin dhuga	
**502**	Dhugaatii alkoolii akka dhugduuf maal si kakaase?	1. Dibbirtii/Depression<br>2. Dhiibbaa hiriyaa<br>3. Beela irranfachuuf<br>4. Soda hir'isuuf (qunnamtii saalaa, hanna)<br>5. Kan biraa, ibsi /_____/	
**503**	Qoricha araadaa fudhattaa?	1. Eeyyee<br>2. Lakki	
**504**	Eeyyee yoo ta'e kam yeroo baay'ee fayyadamtaa?	1. Jimaa qama'uu<br>2. Ganjaa/Hashishii,<br>3. Xamboo aarsuu<br>4. Beenzila urgeeffachuu<br>5. Qorichoota lilmoo<br>6. Kan biraa, ibsi /_____________/	
**505**	Qorichoota fayyadamuuf maaltu si kakaase?	1. Dibbirtii/Depression<br>2. Dhiibbaa hiriyaa<br>3. Beela irranfachuuf<br>4. Soda hir'isuuf (qunnamtii saalaa, hanna)<br>5. Kan biraa, ibsi /_____/	
**Shaakala amaloota hamaa walqunnamtii saalaa fi fayyaa walhormaataa**			
**601**	Walqunnamtii saalaa gootee beektaa?	3. Eeyyee<br>4. Lakki	
**602**	Q601 eeyyee yoo ta'e, umrii meeqatti yeroo dursaaf walqunnamtii saalaa gootee?	_____	
**603**	Maalif yeroo san walqunnamtii saalaa gootee?	1. Heerumee/fuudhen<br>2. Jaaladheen<br>3. Fedhii dhuunfaatif	

		4. Dirqiin gudeeddin<br>5. Maallaqa ykn qarshii argachuuf<br>6. Dhiibbaa hiriyaatin<br>7. Dhiibba jimaa/alkolitiin<br>8. Kan biraa, ibsi /_______/	
**604**	Eenyu waliin walqunnamtii saalaa yeroo dursaa gootee?	1. Haadha manaa/abbaa manaa<br>2. Jaalallee dhaabbataa waliin<br>3. Jaalallee yeroo waliin<br>4. Miseensa maatii waliin<br>5. Daldaltoota salqunnamtii waliin<br>6. Kan biraa, ibsi /_______/	
**605**	Ji'a tokko darbe keessatti walqunnamtii gootee beektaa?	1. Eeyyee<br>2. Lakki	
**606**	Haga hammaa hiriyaa salqunnamtii meeqa qabdaa?	1. _____<br>2. NA	
**607**	Waggaa tokko darbe keessatti fedhii malee gudeedamtee beektaa?	1. Eeyyee<br>2. Lakki	
**608**	Eeyyee yoo ta'e kondomii ykn qusannoo maatii fayyadamtee beektaa?	1. Eeyyee<br>2. Lakki	
**609**	Q608 lakki yoo ta'e, maalif?	3. Karoora malee ture<br>4. Dirqamaan<br>5. Kondomii argachuuf fagoo ture<br>6. Qusannoon maatii hin argamu<br>7. Mi'aa ture<br>8. Walqunnamtii saalaa yeroo hin murtoofnen godha<br>9. Kan biraa, ibsi /_________/	
**610**	Rakkoon walqunnamtii saalaa ykn walhormaata fayyaa waggaa tokko darbe kana keessatti si mudate maalii?	1. Ulfa hin barbaadamne<br>2. Ulfa baasuu<br>3. Walqunnamtii saalaa machii<br>4. Dhukkuboota saal qunnamtii	

		5. Dirqiin gudeeddii<br>6. Yaalii dirqiin gudeeddii<br>7. Homtuu<br>8. Kan biraa, ibsi /_______/	
**611**	Ulfooftee beektaa? [dubartoota qofaaf]	1. Eeyyee<br>2. Lakkii	
**612**	Ulfichi kan hin barbaadamne ture?	1. Eeyyee<br>2. Lakki	
**613**	Lakki yoo ta'e, akkamiin ulfaa'uu dandeessee?	1. Qusannoon maatii hin jiru<br>2. Gudeeddii<br>3. qusannoo maatii hojjachuu didee<br>4. xiyyeeffannoo kennuu dhiisuu<br>5. kan biraa, ibsi /__________/	
**614**	Deessee beektaa?	1. Eeyyee<br>2. Lakki	
**615**	Hanga hammaatti ulfa baaftee beektaa?	1. Eeyyee<br>2. Lakki	
**616**	Q615 eeyyee yoo ta'e, eessatti baaftee?	1. Dhaabbata fayyaa mootummaa<br>2. Kilinika dhuunfaatti<br>3. Mana nama ulfa baasuu<br>4. Ofii kiyyaan<br>5. Kan biraa, ibsi_______	

Galatoomaa

የመረጃ መሰብሰያ መሳሪያዎች

መጠይቅ ኮድ ቁጥር: ______________

የመረጃ አሰባሳቢ ስም: _______________________

የመለያ ቁጥር	ጥያቄዎች	ምላሽ	መግለጫ
**የጥናት ተሳታፊ ማህበራዊ-ስነ-ህዝብ ባህሪዎች**			
**101**	ዕድሜ (በዓመት)	______	
**102**	ፆታ	1. ወንድ<br>2. ሴት	
**103**	የአሁኑ አድራሻ	1. ከተማ<br>2. ገጠር	
**104**	የትውልድ ቦታ	________	
**105**	ጎሳ	1. ኦሮሞ<br>2. አማራ<br>3. ሀረሪ<br>4. ሌላ ፣ ይግለጹ / ____________ /	
**106**	ሃይማኖት	1. ሙስሊም<br>2. ኦርቶዶክስ<br>3. ፕሮቴስታንት<br>4. ሌላ ፣ ይግለጹ / ____________ /	
**107**	አሁን ያለዎት የትዳር ሁኔታ ምንድነው?	. በአሁኑ ጊዜ ያገባ<br>2. በጭራሽ አላገባም<br>3. ተፋታ<br>4. መበለት<br>5. ሌሎች ፣ ይግለጹ / __________________ /	
**የልጁ የትምህርት ዳራ**			
**201**	የትምህርት ሁኔታ	1. በጭራሽ ትምህርት ቤት አይማሩ<br>2. ማንበብ እና መጻፍ ብቻ	

		3. 1 -4 ክፍል<br>4. 5 - 8 ክፍል<br>5. 9 -12 ክፍል	
**202**	በትምህርት ቤት ውስጥ ተመዝግበው የማያውቁ ከሆነ ፣ ትምህርቱን ለመከታተል እንቅፋት የሚሆኑት ነገሮች ምንድን ናቸው?	1. የቤተሰብ ድህነት / የገንዘብ ችግሮች<br>2. በአቅራቢያ ምንም ትምህርት ቤት አይገኝም<br>3. ቤተሰብ ዋጋን ከትምህርት ጋር አላያያዘም<br>4. ቤተሰቡን ለመርዳት ቤት መቆየት ነበረበት<br>5. 5. ሌሎች (ይግለጹ)_______________	
**203**	አሁን ከትምህርት ቤት ማቋረጥ ከሆኑ ትምህርትዎን ለምን አቆሙ?	1. ቤተሰብ ከአሁን በኋላ የትምህርት ቤት ክፍያ መክፈል አልቻለም<br>2. የቤተሰብን ገቢ ለመደጎም መሥራት ነበረብኝ<br>3. ደካማ የትምህርት አፈፃፀም<br>4. የጤና / የህክምና ምክንያቶች<br>5. በባህሪው ምክንያት ተባረዋል<br>6. የራሴን ንባብ ለማድረግ<br>7. ሌሎች (ይግለጹ)________	
**204**	አሁን ትምህርትዎን ለመቀጠል እድል ከተሰጠዎት እድሉን ለመጠቀም ፈቃደኛ እና ደስተኞች ናችሁ?	1. አዎ<br>2. አይ	
**ቃለ-መጠይቅ አድራጊው የቤተሰብ አመጣጥ**			
**301**	ወላጆች አሉዎት?	**1.** አዎ<br>2. አይደለም	
**302**	ወላጆችህ በሕይወት አሉ?	**1.** ሁለቱም በሕይወት አሉ<br>2. ሁለቱም ሞተዋል<br>3. በህይወት ያለች እናት ብቻ<br>4. በህይወት ያለ አባት ብቻ<br>5. አላውቅም	
**303**	ሁለቱም በሕይወት ካሉ የወላጆቻችሁ የጋብቻ ሁኔታ ምን ይመስላል / ነበር?	1. ተጋብቶ አብሮ መኖር<br>2. በመጥፎ ጋብቻ ዝግጅቶች ምክንያት የተፋቱ	

		3. ሁኔታዊ መለያየት (ጦርነት ፣ የሥራ መፈናቀል…)<br>4. መበለት<br>5. ነጠላ ወላጅ<br>6. በሞት ምክንያት ተለያይቷል<br>7. አታውቅም<br>8. ሌሎች (ይግለጹ)________________	
**304**	ከተፋቱ በእርስዎ አመለካከት ለፍቺው መንስኤው ምንድነው?	1. ድህነት<br>2. አባት ፍለጋ ሥራ ፍለጋ ከቤት ወጣ<br>3. መጥፎ ልማድ<br>4. ሌሎች (ይግለጹ)________	
**305**	የእናትዎ መተዳደሪያ ዘዴ ምን / ነበር?	1. መደበኛ ያልሆነ የዕለት ተዕለት ሠራተኛ<br>2. ጥቃቅን ሻጭ / ነጋዴ<br>3. ችሎታ ያለው ሠራተኛ / በራሱ ሥራ የሚሠራ<br>4. የቤት ሰራተኛ<br>5. ለማኝ<br>6. የቤት ሚስት<br>**7.** ሌላ (ይግለጹ)________________	
**306**	የአባትዎ የገቢ / የኑሮ መተዳደሪያ ዘዴ ምን / ነበር?	1. እርሻ<br>2. መደበኛ ያልሆነ የቀን ሰራተኛ<br>3. ነጋዴ<br>4. የመንግስት ሰራተኛ<br>5. የግል ሰራተኛ<br>6. ለማኝ<br>7. ወታደር / x-ወታደር<br>8. ጠባቂ<br>9. ሌላ (ይግለጹ)___________________	
**307**	አሁን ወላጆችህ የት ይኖራሉ?	1. በሐረር<br>2. በሌላ የከተማ ከተማ<br>3. በገጠር አካባቢዎች	

		4. አታውቅም	
**308**	አሁን ቤተሰቡን የሚደግፈው ማነው?	1. አባት ብቻ<br>2. እናት ብቻ<br>3. ሁለቱም እናት እና አባት<br>4. እኔ ራሴ<br>5. ሌላ (ይግለጹ)______________	
**309**	ወላጆችዎ ስደተኞች ከሆኑ የትውልድ ቦታቸውን ለቀው ወጡ?	1. የድርቅ / ረሃብ ሁኔታ<br>2. ሥራ ለመፈለግ<br>3. ለሕክምና ሕክምና<br>4. አታውቅም<br>5. ሌሎች (ይግለጹ)_____________	
**310**	የወላጆችዎ ወርሃዊ ገቢ ምንድነው? በግምት	1. <1000<br>2. 1000-2000<br>3. >2000<br>4. እኔ አላውቅም	
**311**	የአባትዎ የትምህርት ሁኔታ ምንድነው?	1. ማንበብና መጻፍ<br>2. ማንበብ እና መጻፍ ይችላል<br>3. 3. ከፍተኛ ክፍል ተጠናቀቀ	
**312**	ወላጆችዎ የሚኖሩት በምን ዓይነት ቤት ውስጥ ነው?	1. የጡብ ድንጋይ<br>2. የቀርከሃ ዱላዎች<br>3. የጭቃ ቤት<br>4. ፕላስቲክ / ቁራጭ<br>5. ቆርቆሮ<br>6. ሌላ (ይግለጹ) ______________	
**የልጁን የሕይወት ዘይቤ / ሁኔታዎች**			
**401**	ለምን ያህል ጊዜ በጎዳና ሕይወት ውስጥ ተሰማርተሃል? (የጎዳና ህይወት ቆይታ)	1. _____<br>8, አላውቅም / አላስታውስም	
**402**	ወደ ጎዳና አኗኗር እንድትወስድ ያስገደዱህ ነገሮች ምንድን ናቸው?	1. የእኩዮች ተጽዕኖ<br>2. ሥራ ለመፈለግ<br>3. ከወላጆች ጋር በድርድር	

		4. ምግብ ፍለጋ<br>5. በግዳጅ<br>6. የወላጆች ሞት<br>7. በቤተሰብ ውስጥ ሰላም ማጣት<br>8. ቤተሰብ እኔን መደገፍ አለመቻል<br>9. ሌላ ፣ (ይግለጹ) _________	
**403**	ትሰራለህ?	1. አዎ<br>2. አይደለም	
**404**	መልስዎ አይሆንም ከሆነ እንዴት ይተርፋሉ?	_____________	
**405**	የጎዳና ላይ ሥራዎ ምንድነው?	1. መለመን<br>2. የጫማ አንጸባራቂ<br>3. ትናንሽ እቃዎችን መሸከም<br>4. መልዕክቶችን ማድረስ<br>5. አከፋፋይ<br>6. የታክሲ ልጅ<br>7. ሌላ (ይግለጹ) _______________	
**406**	በየቀኑ በአማካይ ስንት ያገኛሉ?	1. ከአምስት ብር በታች<br>2. 5 -10 ብር<br>3. 10 -20 ብር<br>4. 30-50 ብር<br>5. ከ 50 ብር በላይ	
**407**	ዕለታዊ ገቢዎን እንዴት ያጠፋሉ?	1. ምግብ<br>2. መጠለያ<br>3. ጨርቅ<br>4. የትምህርት ቤት ክፍያ<br>5. ቤተሰብን መርዳት<br>6. ሌላ (ይግለጹ)______________	
**408**	ለቤተሰብ በጀቱ አስተዋጽኦ እያደረጉ ከሆነ ለወላጆችዎ ላበረከቱት አስተዋጽኦ ምን አመለካከት አለው?<br>1.	1. ደስተኛ<br>2. ደስተኛ ያልሆነ<br>3. ግዴለሽነት<br>4. 4. አታውቅም	

**409**	የዕለት ገቢዎ አስተማማኝ ነው?	1. አዎ<br>2. አይደለም	
**410**	በጎዳናዎች ላይ ትለምናለህ?	1. አዎ<br>2. አይደለም	
**411**	አዎ ከሆነ ፣ ስንት ጊዜ ነው?	1. ሁል ጊዜ<br>2. ብዙ ጊዜ<br>3. አንዳንድ ጊዜ<br>4. አልፎ አልፎ<br>5. በጭራሽ	
**412**	ወደ ጎዳና ሕይወት ከመጀመርዎ በፊት ከማን ጋር ይኖሩ ነበር?	1. ከሁለቱም ወላጆች ጋር<br>2. ከእናቴ ጋር<br>3. ከአባት ጋር ብቻ<br>4. ከቅርብ ዘመዶች ጋር	
**413**	አሁን ከማን ጋር ነው የምትኖረው?	1. ከእናቴ ጋር<br>2. ከሁለቱም ወላጆች ጋር<br>3. ከዘመዶች ጋር<br>4. ከአባት ብቸኛ ጋር<br>5. ከእኩዮች / ጓደኞች ጋር<br>6. ሌላ፣ ይግለጹ ___________________	
**414**	ጎዳና ላይ ሳሉ ማን ይረዳዎታል እንዲሁም ይጠብቃል?	1. ማንም የለም<br>2. እህትማማቾች<br>3. የእኩዮች ቡድኖች<br>4. ወላጆች<br>5. የቅርብ ዘመድ	
**415**	ብዙውን ጊዜ ቤተሰቦችዎን ምን ያህል ያዩታል / ይጎበኛሉ?	1. በየቀኑ<br>2. በሳምንት ውስጥ ቢያንስ አንድ ጊዜ<br>3. በየወሩ አንዴ<br>4. በዓመት ውስጥ ጥቂት ጊዜ ብቻ<br>5. አልፎ አልፎ<br>6. ባልተስተካከለ ሁኔታ	

		7. በጭራሽ	
**416**	ወላጆችዎ ወይም አሳዳጊዎችዎ ኑሮዎን እና ጎዳናዎች ላይ መሥራትዎን ያፀድቃሉ / አይቀበሉም ብለው ያስባሉ?	1. ማጽደቅ<br>2. አልፈቀደም<br>3. አይጨነቁ<br>4. አታውቅም	
**417**	በቀን ስንት ጊዜ ትበላለህ?	1. አንዴ<br>2. ሁለት ጊዜ<br>3. ሶስት	
**418**	ብዙውን ጊዜ የሚበሉት ምን ዓይነት ምግብ ነው?	_______	
**419**	ብዙውን ጊዜ የት ይተኛሉ?	1. ቤት<br>2. የቤተክርስቲያን ጓሮዎች<br>3. የመስጊድ ጓሮዎች<br>4. የአውቶቡስ ማቆሚያዎች<br>5. በረንዳ ላይ<br>6. ርካሽ ሆቴሎች	
**420**	በዕለት ተዕለት የጎዳና ሕይወትዎ ውስጥ በአሁኑ ጊዜ የሚያጋጥሙዎት ዋና ዋና ችግሮች ምንድናቸው?		
**421**	በጎዳናዎች ላይ ሲኖሩ ወይም ሲሰሩ ታምመው ያውቃሉ?	1. አዎ<br>2. አይደለም	
**422**	አዎ ለ Q421 ከሆነ ምን ዓይነት ህመም ነው?		
**423**	እንደታመሙ ብዙውን ጊዜ ማንን ነው የሚደውሉት?		
**424**	ማንኛውም ዓይነት ልማድ / ሱሰኛ አለዎት?	1. አዎ<br>2. አይደለም	
**425**	ለ Q424 አዎ ከሆነ ምን ዓይነት?		
**426**	በጎዳናዎች ላይ በሚኖሩበት / በሚሰሩበት ጊዜ በአዋቂዎች ተደብድበው ወይም ተጎድተው ያውቃሉ?	1. አዎ<br>2. አይደለም	
**427**	ማንኛውም ወሲባዊ ድርጊት እንድትፈጽም ያስገደደዎት ሰው አለ?	1. አዎ	

		2. አይደለም	
**428**	ለገንዘብ ሲሉ ከአንድ ሰው ጋር የግብረ ሥጋ ግንኙነት ፈጽመው ያውቃሉ?	1. አዎ<br>2. አይደለም	
**429**	ከሚከተሉት ውስጥ የትኛው አስቸኳይ ፍላጎትዎ ነው?	1. ምግብ<br>2. ልብስ<br>3. መጠለያ<br>4. ትምህርት<br>5. የቤተሰብ ድጋፍ<br>6. የገንዘብ ድጋፍ<br>7. እንደገና ተቀላቀል<br>8. ሌላ (ይግለጹ)_____________	
**430**	የአኗኗር ዘይቤዎን ለማሻሻል እንዲረዳዎ በአሁኑ ጊዜ መከናወን ያለበት በጣም ተገቢው ጣልቃ-ገብነት እርምጃ / ግላዊነት በግልዎ ምን ይመስልዎታል ወይም ያምናሉ?		
**ንጥረ ነገር እና አልኮል አላግባብ መጠቀም**			
**501**	እንደ ጠላ ፣ ጠጅ ፣ ቢራ ፣ አረኪ እና የመሳሰሉት የአልኮል መጠጥ ይጠጣሉ?	1. በጭራሽ አልጠጡም<br>2. አንዴ ወይም ሁለቴ ሞክሬያለሁ<br>3. ብዙ ጊዜ እጠጣለሁ<br>4. በየቀኑ እጠጣለሁ	
**502**	አልኮል እንዲጠቀሙ ምን ያስነሳዎታል?	1. ድብርት<br>2. የእኩዮች ተጽዕኖ<br>3. ረሃብን ለመጠበቅ<br>4. ፍርሀትን ለመጠበቅ (ወሲብ ፣ መስረቅ)<br>5. ሌላ ፣ ይግለጹ / ______ /	
**503**	አደንዛዥ ዕፅ ይጠቀማሉ?	1. አዎ<br>2. አይደለም	
**504**	አዎ ከሆነ የትኛው መድሃኒት ብዙ ጊዜ ይጠቀማሉ?	1. የቻት ማኘክ<br>2. ጋንጃ / ሀሺሽ ፣<br>3. ሲጋራ ማጨስ	

		4. ሳንፍፍ ቤንዜን<br>5. በመርፌ የሚሰጥ መድሃኒት<br>6. ሌላ ፣ ይግለጹ / _____________ /	
**505**	አደንዛዥ ዕፅን እንዲጠቀሙ ምን ያስነሳዎታል?	1. ድብርት<br>2. የእኩዮች ተጽዕኖ<br>3. ረሃብን ለመጠበቅ<br>4. ፍርሀትን ለመጠበቅ (ወሲብ)<br>5. ሌላ ፣ ይግለጹ /________________/	
**አደጋ ተጋላጭ ወሲባዊ እና ተዋልዶ ጤና ልምዶች**			
**601**	የግብረ ሥጋ ግንኙነት ፈጽመው ያውቃሉ?	1. አዎ<br>2. አይደለም	
**602**	ለቁ 601 አዎ ከሆነ በመጀመሪያ የግብረ ሥጋ ግንኙነት የፈፀሙበት ዕድሜ ስንት ነው?	______	
**603**	ለመጀመሪያ ጊዜ የግብረ ሥጋ ግንኙነት ለመፈፀም ለምን ወሰኑ?	1. አገባለሁ<br>2. በፍቅር ወድቀዋል ፡፡<br>3. የግል ፍላጎት.<br>4. ማስገደድ (አስገድዶ መድፈር) ፡፡<br>5. ገንዘብ እና ሌሎች ስጦታዎች ለማግኘት.<br>6. የእኩዮች ተጽዕኖ<br>7. የጫት / የአልኮሆል ተጽዕኖ<br>8. ሌሎች ፣ ይግለጹ / ________ /	
**604**	የመጀመሪያ የግብረ ሥጋ ግንኙነትዎን ከማን ጋር አደረጉ?	1. ባል / ሚስት<br>2. ከተረጋጋ ወንድ / ሴት ጓደኛ ጋር<br>3. ከተለመደው ወንድ / ሴት ጓደኛ ጋር<br>4. ከቤተሰብ አባል ጋር<br>5. ከንግድ ወሲባዊ ሠራተኛ ጋር<br>6. ሌሎች ፣ ይግለጹ / ________ /	
**605**	ባለፈው 1 ወር ውስጥ የግብረ ሥጋ ግንኙነት ፈጽመዋል?	1. አዎ<br>2. አይደለም	

**606**	ስንት የወሲብ አጋሮች አጋጥመው ያውቃሉ?	3. ______<br>4. NA	
**607**	ያለፉት 12 ወራት ያለእርስዎ ፈቃድ ተደፍረዋል?	1. አዎ<br>2. አይደለም	
**608**	አዎ ከሆነ ፣ ኮንዶም ወይም የእርግዝና መከላከያዎችን ተጠቅመዋል?	1. አዎ<br>2. አይደለም	
**609**	አይደለም፣ ለምን አይሆንም?	1. ያልታቀደ ነበር<br>2. የቫዮሌት ተቃራኒ<br>3. ኮንዶምን ለማግኘት በጣም ወይም<br>4. የእርግዝና መከላከያ (ተደራሽ አይደለም)<br>5. ዘዴው ውድ ነበር<br>6. አልፎ አልፎ ወሲብ አለብኝ ፡፡<br>7. ሌላ ፣ ይግለጹ / __________ /	
**610**	ባለፉት 12 ወራት ውስጥ ምን ዓይነት ወሲባዊ እና ሥነ ተዋልዶ ጤና ችግሮች አጋጥመውዎታል?	1. የማይፈለግ እርግዝና<br>2. ፅንስ ማስወረድ<br>3. አልኮሆል ጤናማ ያልሆነ ወሲብ<br>4. የአባለዘር በሽታዎች<br>5. ማስገደድ (አስገድዶ መድፈር) ፡፡<br>6. ማስገደድ (አስገድዶ መድፈር) ሙከራ አድርጓል<br>7. ችግር የለም<br>8. ሌላ ፣ ይግለጹ / ________ /	
**611**	እርጉዝ ነዎት? [ለፍልስፍናዎች ብቻ]	1. አዎ<br>2. አይደለም	
**612**	እርግዝና ይፈለጉ ነበር?	1. አዎ<br>2. አይደለም	
**613**	አይሆንም ከሆነ እንዴት አረገዙ?	1. የእርግዝና መከላከያ ዘዴ አይገኝም<br>2. ማስገደድ (አስገድዶ መድፈር)<br>3. ዘዴ አለመሳካት<br>4. አፅንዖት ማጣት<br>5. ሌላ ፣ ይግለጹ / ___________ /	

**614**	ወለዱ?	3. አዎ<br>4. አይደለም	
**615**	ፅንስ ማስወረድዎን ያውቃሉ?	3. አዎ<br>4. አይደለም	
**616**	ለ Q615 አዎ ከሆነ የት ነው ያስወገደከው?	1. በሕዝብ ጤና ተቋም<br>2. በግል ክሊኒክ<br>3. በፅንስ ማስወገጃ ቤት ውስጥ<br>4. እኔ እራሴ አነሳሁት<br>5. ሌሎች ፣ ይግለጹ…	

አመሰግናለሁ
