# Supplementary material for: Health status of street children and reasons for being forced to live on the streets in Harar, Eastern Ethiopia. Using mixed methods
Source: PLoS One. 2022 Mar 18;17(3):e0265601. doi: 10.1371/journal.pone.0265601 (PMC8932583; doi:10.1371/journal.pone.0265601)
Supplement: S2 File — (PDF) [file pone.0265601.s003.pdf]

Key Informant Interview Guide, 2021

I: Observer /interviewer name________________________Sign _________

II: Location

Region _______ Zone _________ Woreda ___________Kebele _________

Date of discussion _______/________/2021

Introduction

My name is __________________________________. I am working in Haramaya University College of Health and Medical Science (CHMS) and currently collecting the data required for the study conducted by CHMS staff members. We are conducting a survey on the determinant factors and life style of street children living in Harari regional state, Eastern Ethiopia. The information we collect will help the government and partners to plan appropriate strategies important to solve the problems particularly related to street children.

You will not be benefited personally from taking part in this interview. Participating in this study has no risks for you. Neither your name nor the names of any other workers who participated in this study will be included in the dataset or in any reports. You may refuse to answer any question or choose to stop the interview at any time.

In order to capture all of the information that you are about to share with us, we will record the interview using this (show recorder) audio recorder. The audio records will be deleted after complete transcription of the interview on a paper.

N.B: Please, here, the provided questions will be used as a guide, you can add more questions depending on the participants response, probe…

General Question Section	Main Questions and Probe….
us/me few things about yourself, and your role?	

- **What is the children protection related issues in the region?**

Probe:

- What are the priority child protection issues within this community?

- What are the root causes of the priority child protection issues?
- How are child protection laws or regulations implemented in the community?
- What are the barriers to effective implementation of child protection laws or regulations?
- What is being done to prevent abuse, exploitation, or neglect in the community?
- Are there formal or informal mechanisms of support for households that are identified as being at high risk of child abuse or exploitation?
- Do relevant formal and informal authorities have the capacity to perform their roles for prevention and response to child abuse, neglect, and exploitation?
- Do service providers such as teachers, health workers, police, social workers, and counsellors have the capacity to identify, report, and respond to cases of abuse, violence, and exploitation according to their roles? If not, why?
- Is there a mechanism which brings together the different stakeholders and duty bearers for preventing and responding to child abuse, neglect, and exploitation?
- What are the linkages (and gaps) between informal and formal parts and actors in the system?
- Are the social services aimed at vulnerable children and their families monitored by the government and provided in alignment with national standards?

❖ **Tell me a little bit about the issues related to street children?**

Probe: -

- Are there any activities planned or done to reintegrate and reduce the harmful behavior related to street children?
- How and when it was organized?
- What are the criteria you have used (how)?
- Who participated in the program? (From the federal, regional, woreda, etc.)
- Did the street children participate in the program? (Number,
- Community participation in the program?
- Is there any training prepared and given for street children or their families? Can you tell me more about it?

❖ **What are the challenges/ barriers you have faced to reintegrating and reducing the harmful behavior related to street children?** Probe: -

- Technical related factors, financial/budget, experts (human resources), methodological, time-related factors, etc.

- **What do you suggest to solve or minimize these challenges or barriers?**

Probe: -

- What are the concerned organized and issues (specify...) should do to solve the challenges hindering the work on street children?
- What is expected of regional government sectors, woredas, non-governmental organizations (NGOs), and private sectors, among others, to address these challenges?

- **If you haven't did nothing to reintegrate and to reduce the harmful Behaviour related to street children:**

Probe:

- What hindered you to work on this area? (Low concern, resources (financial, human, social and political conditions etc.
- Have you planned to do in the futures? Tell me more about it.
- What do you suggest doing to solve these problems?
- Which the concerned organization (specify) should do to solve these problems?
- What is expected of the government (federal, regional, etc.), non-governmental organizations (NGOs), and community members to solve these problems?

- **Do you have a mechanism to assess the life conditions and determinants factors regarding street children?**

Probe: -

a. Risk assessment,
b. Health related survey,
c. etc.

- **Have you ever conducted survey or received feedback regarding street children life conditions and determinants factors?**

Probe: -

a. What was the outcome or feedback?
b. What did you do in response to the outcome or feedback?

c. What do you recommend to improve street children life conditions?

❖ **Community attitudes**

a. Towards street children?
b. Substance use?
c. Sexual and reproductive health?
d. Others (add more….)

❖ **Common health problems among the street children**

a. What are the common health problems among street children?
b. How do these problems differ from the problems experienced by homeless children?
c. What factors contribute to these differences?

❖ **What are services available for the street children?**

Probe: -

- Are there children who have less access to services like food distributions, educational and recreational activities, and health care?
- Are reproductive health services available for street children?
- Are contraceptives and condoms easily available to street children?
- Is testing for HIV and other sexually transmissible diseases (STDs) offered for street children?
- Is care and support available for street children who are infected with HIV and other health problems?
- Are there special considerations for street children related to education?
- Who gave the service?
- What are the potential barriers to the use of this service by street children?
- How have you overcome such barriers?

❖ **Is there any support the street children have got from you?**

Probe:

a. Financial related support, health service, materials, etc

❖ **Can you give me information about sexually transmissible diseases related to street children?**

Probe:

a. Prevalence of STI.

b. How common are psychiatric illnesses among street children?

c. How common are accidents and other trauma among street children?

- **What interventions have worked to reintegrate street children, or failed? (this information is critical for designing interventions).**

Probe:

a. Types of interventions

b. Time

c. Duration

d. Location

e. Number of participants participated

f. Sustainability of the intervention

g. Whom gave/intervene, (skills etc.) (you can add more….)

- **Do you think the number of sexual violence incidents has increased?**

Is there anything you want to add and you believe that it is important?

Thank you
