# Supplementary material for: Health status of street children and reasons for being forced to live on the streets in Harar, Eastern Ethiopia. Using mixed methods
Source: PLoS One. 2022 Mar 18;17(3):e0265601. doi: 10.1371/journal.pone.0265601 (PMC8932583; doi:10.1371/journal.pone.0265601)
Supplement: Attachment — Submitted filename: Reviewer Suggestions.docx [file pone.0265601.s005.docx]

The research on Health Conditions of Street Children, Their General Life Conditions, and Reasons Forcing to Street Life in Harar, Eastern Ethiopia. Using Mixed Methods, is important in the context of Ethopia. However, I have few suggestions and queries on this manuscript.

Title: Title needs to be rephrased by putting main variables in it.

Abstract: “As it stands now little is known about the extent of the problem including health conditions”. This sentence is unclear, what does it means by “extent of the problem”. In the method you didn’t mention about the type of mixed method used in this study. I don’t see 30 days in February in any year. Please recheck the time duration for data collection. In abstract you are not supposed to write the criteria for the significance level, you can report this in main document. “Overall 220 street children were involved in the study. The most common reason that forced the children to resort to a street way of life is to look a job and quarreled with parents.” These first two lines must be included in the method instead of results. I would suggest you to include both qualitative and quantitative results separately in the result section. In the conclusion, you have reported results, which are redundant to what you have written in result, it is suggested to re write the conclusion of abstract.

Introduction:

The problem was not addressed in a convincing manner. In the introduction section, I couldn’t understand the need to conduct research. It would be better if you explicitly write about the literature, policy, and knowledge gaps in the introduction. In the last paragraph of introduction, authors claim “Now days it is being observed the number of street children is significantly increasing where, a little is known about the extent of the problem including factors leading to being street children and their health status”, without any reference. I also recommend to clearly state the objectives of the study.

Method:

Cross-check the dates of data collection “1-30 February, 2021”. It would be better if you mention the date either in abstract or in methodology, avoid repetition.

Study Design and population: I couldn’t find any description or rationale of choosing mixed method and which mixed method has been chosen. You need to specify research questions identifying quantitative and qualitative method. No information about the sample for qualitative study is reported in this section.

Sample size determination and sampling procedure

What does it means by “Out of this various sit”?

Please provide the reference that the higher number of street children are present in the areas which you have selected for the study. Provide the rationale for applying qualitative method and selection of sample for the qualitative study.

Data collection methods and data collection procedure:

Provide the name of the questionnaires used for the quantitative data collection. If you adapted, then explain the steps/ models used to adapt or contextualize the questionnaire. Also report the reliability and validity of the questionnaires. For qualitative study explain what type of interview guideline was developed and why (e.g., structured, semi-structured, or unstructured). Please share few questions from the interview guideline.

In an article you do not need to provide operational definition however, you can provide the rationale for using specific variables through literature in introduction section.

Data Analysis

What is KII??

Explain triangulation in this study specifically, what is the purpose of triangulation and how would you analysed it?

Statistical Analysis

You have applied multivariate, but you didn’t mention whether the data met assumptions for the multivariate analysis. For qualitative, explain how you develop themes, what procedure or model you followed for finalizing the main themes.

The verbatims stated are not intact. One sentence of the verbatim is not connected with the other sentence. Detail explanation of the verbatim is required. When you clarify the type of mixed method, you will be able to integrate the information from the two studies. Otherwise, the analysis section seems disorganized. Interpretation of the results are also not clear.

In Table 4 “Health status and substance use among study participants in Harar city, Ethiopia”, you have mentioned about depression, do you really think that street children understand what is depression?

Discussion

Instead of reporting percentages in results and discussion, it would be very helpful for the reader to understand if interpretation of the results along the supporting or refuting by previous literature would be reported.

I would suggest that discussion must be organized by giving; 1. argument from literature and culture context, 2. implication of the study and then 3. limitations.
