# Supplementary material for: Health status of street children and reasons for being forced to live on the streets in Harar, Eastern Ethiopia. Using mixed methods
Source: PLoS One. 2022 Mar 18;17(3):e0265601. doi: 10.1371/journal.pone.0265601 (PMC8932583; doi:10.1371/journal.pone.0265601)
Supplement: Attachment — Submitted filename: Response to reviwers.docx [file pone.0265601.s006.docx]

**Point by point response to editor and reviwers**

**Title**: **Health Status of Street Children and Reasons for Being Forced to Live on the Streets in Harar, Eastern Ethiopia. Using Mixed Methods**

**Manuscript ID:** PONE-D-21-13888

**From**: Authors

**To**: The editor in chief, PLOS ONE

**Version**: I

**Data**: 25/11/2021

**Subject: Revision of the manuscript**

We appreciate the reviewers' detailed and comprehensive comments. We found the comments to be very helpful, and we appreciate the time and thought that each person put into their constructive comments. We are well aware of the time ,commitment required to provide good reviews and applaud the reviewers for their efforts. We thoroughly revised the paper and responded in detail to the reviewers' questions and comments.The point-by-point description of the changes is provided below.

**For editors**

Comment 1: Please ensure that your manuscript meets PLOS ONE's style requirements, including those for file naming.

*Response 1: thank you for your comment. Now the revised version is edited based on PLOS ONE's style requirements.*

2. Please include additional information regarding the survey or questionnaire used in the study and ensure that you have provided sufficient details that others could replicate the analyses. For instance, if you developed a questionnaire as part of this study and it is not under copyright more restrictive than CC-BY, please include a copy, in both the original language and English, as Supporting Information. If the original language is written in non-Latin characters, for example, Amharic, Chinese, or Korean, please use a file format that ensures these characters are visible."

*Response 2: Thank you very much. We have now added more information about the survey.*

3. Please state whether you validated the questionnaire before testing on study participants. Please provide details regarding the validation group within the methods section."

*Response 3: Thank you for your input. It has been corrected and is now part of the revised document.*

4. Please include a copy of the interview guide used in the study, in both the original language and English, as Supporting Information, or include a citation if it has been published previously."

*Response 4: Thank you. We now include it as an addendum.*

5. You indicated that you had ethical approval for your study. In your Methods section, please ensure you have also stated whether you obtained consent from parents or guardians of the minors included in the study or whether the research ethics committee or IRB specifically waived the need for their consent.

*Response 5: Thank you for your input. It has been corrected and is now part of the revised document. Consent was obtained from the minors' parents or guardians..*

6. In your Data Availability statement, you have not specified where the minimal data set underlying the results described in your manuscript can be found. PLOS defines a study's minimal data set as the underlying data used to reach the conclusions drawn in the manuscript and any additional data required to replicate the reported study findings in their entirety. All PLOS journals require that the minimal data set be made fully available.

*Response 6: Thank you for your comment. It is corrected and incorporated into the revised document.*

7. Your ethics statement should only appear in the Methods section of your manuscript. If your ethics statement is written in any section besides the Methods, please delete it from any other section.

*Response 7: Thank you for your comment. It is corrected and incorporated into the revised document. Ethics statement was omitted from declaration section.*

8. Please include a separate caption for each figure in your manuscript

*Response 8: We are intrigued by your comment, and we accept it; it has been corrected and incorporated into the revised document.*

**For reviewer 1**

Comment 1. The research on Health Conditions of Street Children, Their General Life Conditions, and Reasons Forcing to Street Life in Harar, Eastern Ethiopia. Using Mixed Methods is important in the context of Ethiopia. However, I have a few suggestions and queries on this manuscript

*Response 1: thank you for your comment. The significance of this study in the context of Ethiopia is obvious for a variety of reasons, which are briefly discussed below. I believe it is critical because numerous factors lead a person to leave his or her home and join the life of a street person. It is critical because not everyone in Ethiopia lives on the street; if that were the case, we would not need to study because everyone was affected by these serious issues. So, to mitigate the problems and devise solutions that will undoubtedly address a long-standing issue, this research is crucial.*

Comment 2. Title: The title needs to be rephrased by putting main variables in it.

*Response 2: thank you for your nice comment. It is corrected as the Health Status of Street Children and Reasons for Being Forced to Live on the Streets in Harar, Eastern Ethiopia. Using Mixed Methods.*

Comment 3. “As it stands now little is known about the extent of the problem including health conditions”. This sentence is unclear, what does it mean by “extent of the problem”.

*Response 3: thank you for your comment and questions. When we talk about the extent of the problem, we're referring to the prevalence of Streetism, which includes health conditions. This sentence can be paraphrased as “Currently, little is known about the prevalence of the Streetism, including health conditions.*

Comment 4. In the method, you didn’t mention the type of mixed method used in this study.

*Response 4: We are intrigued by your comment, and we accept it; it has been corrected and incorporated into the revised document. The study designs were quantitative cross-sectional studies and qualitative phenomenological studies.*

Comment 5. I don’t see 30 days in February in any year. Please recheck the time duration for data collection

*Response 5: Thank you for your comment and question. We apologize for such kind of error. Now it is corrected and incorporated in the revised manuscript.*

Comment 6. In the abstract you are not supposed to write the criteria for the significance level, you can report this in the main document

*Response 6: Thank you for your comment and suggestion. It is corrected and incorporated in the revised manuscript.*

Comment 7. Overall, 220 street children were involved in the study. The most common reason that forced the children to resort to a street way of life is to look for a job and quarreled with parents.” These first two lines must be included in the method instead of results.

*Response 7: Thank you for your insightful and wonderful comment. We accept your comment and we incorporate it into the revised manuscript.*

Comment 8. I would suggest you include both qualitative and quantitative results separately in the result section.

*Response 8: Thank you for your comment. We accept it and corrected it in the revised manuscript.*

Comment 9. In the conclusion, you have reported results, which are redundant to what you have written in the result, it is suggested to rewrite the conclusion of the abstract.

*Response 9: Thank you for your specific comment. We accept it and corrected it in the revised manuscript.*

Comment 10. The problem was not addressed convincingly. In the introduction section, I couldn’t understand the need to conduct research. It would be better if you explicitly write about the literature, policy, and knowledge gaps in the introduction.

*Response 10. Thank you for your insightful comment. We revised the introduction section based on your recommendations.*

Comment 11. In the last paragraph of the introduction, the authors claim “Nowadays it is being observed the number of street children is significantly increasing were, a little is known about the extent of the problem including factors leading to being street children and their health status”, without any reference.

*Response 11. Thank you for your specific comment. We revised the citation. Now it is cited and a change was made in the revised manuscript.*

Comment 12. I also recommend stating the objectives of the study.

*Response 12. Thank you for your wonderful and eye-catching comments. We accept it and revise accordingly.*

Comment 13. Cross-check the dates of data collection “1-30 February 2021”.

*Response 13: Thanks for your eagle eyes we are already changed it*

Comment 14. It would be better if you mention the date either in the abstract or in methodology, avoiding repetition.

*Response 14: thank you for your insightful suggestion. Now the revised version is edited and corrected.*

Comment 15: I couldn’t find any description or rationale for choosing a mixed-method and which mixed method has been chosen.

*Response 15: thank you for your comment. Bringing together qualitative and quantitative research and data in a single study. Mixed-method research is a hybrid that emerged from the most appropriate combination of quantitative and qualitative research methods for a research project. It aimed to maximize the strengths and minimize the weaknesses of the two research methods, increasing the validity of research findings and providing other benefits. As a result, we attempted to use qualitative and quantitative research methods to improve the validity and credibility of the results in particular, as well as the overall research in general. To elaborate further, we employ a qualitative study design based on the Phenomenological approach that aims to develop a complete, accurate, clear, and articulate description and understanding of a particular human experience or experiential moment (Schacht, 1972).*

Comment 16: You need to specify research questions identifying quantitative and qualitative methods.

*Response 16: thank you for your comment. It is corrected and incorporated in the revised manuscript.*

Comment 17: No information about the sample for the qualitative study is reported in this section.

*Response 17:* *Thank you for your comment. The sample size was determined based on the level of information saturation and the variety of ideas among the sub-groups. Data saturation involves sampling until no new information is obtained and redundancy is achieved. We conducted twelve focus group discussions (FGDs) with six participants from various groups and 6 key informants.*

Comment 18: What does it mean by “Out of this various sit”?

*Response 18: Thank you for your question and comment. This means that of the seven areas mentioned... we attempted to paraphrase the sentences containing this phrase as follows. Street children can be found in a variety of locations throughout Harar. Seven are chosen on purpose because there is a higher concentration of street children in these areas of town known as Arategna, Ajip, Canal, Botte, Shewa ber, Feres Megala, and Andegan Menged. This was accomplished through observation and consultation with key informants working with street children.*

Comment 19: Please provide the reference that the higher number of street children are present in the areas which you have selected for the study.

*Response 19: Thank you for your comment. This was the data source obtained from the social affairs office*

*Table 1: Distribution of sample interviewees by selected sites in the Harar city*

| *S. No* | *Name of the Sub town* | *Total of the registered population* | *Number of the interviewees* |
| --- | --- | --- | --- |
| *1* | *Arategna* | *50* | *23* |
| *2* | *Agip* | *61* | *28* |
| *3* | *Canal* | *53* | *24* |
| *4* | *Bote* | *92* | *42* |
| *5* | *Shewa Ber* | *101* | *46* |
| *6* | *Andegan Menged* | *99* | *44* |
| *7* | *Feres megala* | *46* | *21* |
|  | *Total* | *502* | *228* |

Comment 20: Provide the rationale for applying the qualitative method and selection of sample for the qualitative study.

*Response 20: Thank you for your comment and suggestion. There are plenty of rationale for applying the qualitative method. To mention a few of them, to obtain more in-depth information on cases (subjective), to validate and confirm the information gathered, and to increase the validity of research findings. It is useful in establishing meaningful relationships between the researcher and ordinary people, which allows for a better understanding of their world and interpretation of the results in terms of a qualitative approach. So, an attempt was made to apply qualitative methods to study these vulnerable groups. Regarding the selection of the sample, participants were selected based on criteria based purposive sampling to get important information from lived experience.*

Comment 21: Provide the name of the questionnaires used for the quantitative data collection. If you adapted, then explain the steps/ models used to adapt or contextualize the questionnaire.

*Response 21: Dear Reviewer, when adapting the questioners, we kept three things in mind: Language, Pretesting, and the specific question that will be modified*

*Steps one:* *We translated it while keeping the purpose of the questionnaire and the intent of the questions in mind. It was done by group members who speak both languages fluently translate it. To ensure the translation's accuracy, the questionnaire was translated back into English by someone who had not seen the original version and was unfamiliar with the questionnaire's context. The back-translated version is then compared to the original, and any meaning differences are corrected.*

*Step two: To ensure a cross-validity we tried to interview a set of respondents in English and another set in the local language such as Afaan Oromo and Amharic, their answers were then compared to detect differences in understanding.*

*Step three: Pretesting: The goals of pre-testing are well known: to identify questions that are poorly understood, ambiguous, or elicit hostile or other undesirable responses. We attempted to conduct a pretest using the already-translated questionnaire. We tried to implement all the steps in pretesting such as obtaining an evaluation of a questionnaire and testing the revised questionnaire through its paces on friends, colleagues, and so on.*

Comment 22: Also report the reliability and validity of the questionnaires.

*Response 22: When choosing a survey instrument, reliability and validity must be taken into account. The consistency with which an instrument produces the same results across multiple trials are referred to as its reliability. The degree to which an instrument measures what it was designed to measure is known as its validity. Statistically,* *we performed Cronbach's Alpha, which is a measure used to assess the quality of our employed instruments. The result was 0.87, which was within acceptable ranges.*

Comment 23: For qualitative study explain what type of interview guideline was developed and why (e.g., structured, semi-structured, or unstructured). Please share a few questions from the interview guideline.

*Response 23: Thank you for your comment. In our study, a semi-structured interview guideline was used. Dear reviewers, in response to your humble request, please find below a few questions from the interview guideline.*

- *What relationship does your organization/office have with the children of the street?*
- *To what extent is the government concerned about the situation of the street children?*
- *What are the main problems that they face?*
- *What services that your office currently offering to the street children?*

Comment 24: In an article, you do not need to provide an operational definition however, you can provide the rationale for using specific variables through literature in the introduction section.

*Response 24: Dear reviewer, we gratefully received your constructive suggestion and omitted it (operational definition) from an article.*

Comment 25: What is KII??

*Response 25: Dear reviewers, please accept our apologies for such a clumsy error when we say KII we mean key informant interview!!*

Comment 26: Explain triangulation in this study specifically, what is the purpose of triangulation and how would you have analyzed it?

*Response 26: Dear Respected Reviewer, as we all know, triangulation is a well-known technique for increasing the credibility and validity of research findings. Triangulation is a research study can help to ensure that fundamental biases caused by the use of a single method or observer are overcome by combining theories, methods, or observers. In our study, we attempted to use this robust method to improve the trustworthiness and accuracy of our findings. As a result, methodological (both qualitative and quantitative methods) and data triangulation (from different sources using different data collection methods) were carried out to improve the credibility and validity of the research findings*

Comment 27: You have applied multivariate, but you didn’t mention whether the data met assumptions for the multivariate analysis.

*Response 27: Thank you for your constructive comment. Dear reviewer, before performing a multivariate regression analysis, we verified that all assumptions and data met the criteria, such as*

- *The relationship between independent variables and dependent variables was linear (which was checked by producing the scatter plots)*
- *There was no multicollinearity in our data (on collinearity diagnosis we tried to see VIF standard error and Tolerance score)*
- *Values of the residuals were found normality distributed (Tested by looking at P-P plots from the model)*
- *A variance of the residuals was found constant (which was checked by producing the scatter plots)*
- *No influential cases were biasing our models (checked by looking at Cooks distance)*

Comment 28: For qualitative, explain how you develop themes, what procedure or model you followed for finalizing the main themes.

*Response 28: thank you for your comment. We follow the inductive thematic analysis procedures. First deeply read and read again and again. Then we code the ideas. Then we categorize similar ideas and then it was developed into their thematic areas. To do this we carefully examine the data to identify common themes – topics, ideas, and meaning patterns that appear repeatedly. To finalize the main themes, we followed the procedures such as familiarization, coding, generating themes, reviewing themes, defining and naming them, and writing up.*

Comment 29: The verbatims stated are not intact. One sentence of the verbatim is not connected with the other sentence. Detail explanation of the verbatim is required.

*Response 29: Thank you for your comment and suggestion. It is corrected and incorporated in the revised manuscript.*

Comment 30: When you clarify the type of mixed-method, you will be able to integrate the information from the two studies. Otherwise, the analysis section seems disorganized. Interpretation of the results is also not clear.

*Response 30: Thank you for your comment. It is corrected and incorporated into the revised manuscript.*

Comment 31: In Table 4 “Health status and substance use among study participants in Harar city, Ethiopia”, you have mentioned depression, do you think that street children understand what is depression?

*Response 31: Dear Reviewer, yes, they have no idea what depression entails. We asked them in their native language about depression; they had no idea about the scientific terms or nomenclatures of depression that here we used to report to the scientific world.*

Comment 32: Instead of reporting percentages in results and discussion, it would be very helpful for the reader to understand if the interpretation of the results along the supporting or refuting by previous literature would be reported

*Response 32: Thank you for your comment. Now it is modified based on the comment provided.*

Comment 33: I would suggest that discussion must be organized by giving; 1. argument from literature and culture context, 2. implication of the study, and then 3. limitations.

*Response 33: Thank you for your comment. It is corrected and incorporated in the revised manuscript.*

**For reviewer 2**

Comment 1. The title is required to be re-phrased

*Response 1. Thank you for your specific comment. It is corrected and incorporated into the revised manuscript.*

Comment 2.  Table 1: Under father’s educational status, it is mentioned that around 21% of the children have fathers with education level “highest grade completed”. This category needs to be defined clearly.

*Response 2. thank you for your specific comment. We accepted the comment and it is corrected in the revised document. According to our study, this is to mean that from grade secondary level (8-10) to preparatory level (10-12)*

Comment 3. Line 226-232: No need to report frequency since percentages are already given.

*Response 3. Thank you for your nice comment. We incorporate comments in the revised manuscript.*

Comment 4. A thorough spelling and grammar check is required for the entire manuscript.

*Response 4. Thank you for this comment. It is corrected and incorporated in the revised manuscript.*

Comment 5. This paper can be substantially improved by adding more information from the focused group discussions. The qualitative part of the study needs more attention from the authors.

*Response 5. Thank you for your insight full comment. It is corrected on the revised document.*

Thank you
