# Supplementary material for: Health status of street children and reasons for being forced to live on the streets in Harar, Eastern Ethiopia. Using mixed methods
Source: PLoS One. 2022 Mar 18;17(3):e0265601. doi: 10.1371/journal.pone.0265601 (PMC8932583; doi:10.1371/journal.pone.0265601)
Supplement: Attachment — Submitted filename: Second Review.docx [file pone.0265601.s007.docx]

**Health status of street children and reasons for being forced to live on the streets in Harar, Eastern Ethiopia. Using mixed methods**

This research is important in the context of Ethopia and much needed in current scenario. What and how this research adds in the literature and help policy maker to work for the betterment of street children. I can see improvement in the manuscript, but I have few comments and suggestion for your manuscript, which are as follows:

**Abstract**

1. Introduction is well written
2. In Method section, you need to report type of mixed method (e.g., sequential or parallel). I have few questions about phenomenological design (See method section).I want to know the reason for conducting interview for quantitative research design and for qualitative you have done focus group. There is no need to mention softwares in abstract.
3. Results: You didn’t mention result heading. For abstract you can explain results by integrating findings of quantitative and qualitative results.
4. Conclusion is good.

**Main Manuscript**

Introduction:

You have explained the problem in the introduction, but you can add literature from the similar context or from the courtiers which have similar culture and context, to explain causes that force street children to stay on streets.

Material and Method

1. Study setting, and period is explained quite well

2. Explain type of mixed method design. In case of phenomenological study, you need to explain the lived experience. What experience you are studying and how.

3. Sample size determination is fine for quantitative study but for qualitative specially for phenomenological studies the sample size needs to be selected carefully.

4. You didn’t mention name of questionnaires or scales for the quantitative study. Which instrument you adapted. You also have to mention questions of the interview guideline for the focus group.

Results:

This section is quite improved; however, I couldn’t understand the integration point for both qualitative and quantitative studies. Specially, if you are reporting that you did phenomenological study, you need to report lived experiences of the street children.

Discussion

This study has rich data on health status of street children that need to be explained and interpreted in detail. There is multiple limitation for the study that you need to report. You need to explain the future recommendation as well.
