# Supplementary material for: Health status of street children and reasons for being forced to live on the streets in Harar, Eastern Ethiopia. Using mixed methods
Source: PLoS One. 2022 Mar 18;17(3):e0265601. doi: 10.1371/journal.pone.0265601 (PMC8932583; doi:10.1371/journal.pone.0265601)
Supplement: Attachment — Submitted filename: Response to reviwers.docx [file pone.0265601.s008.docx]

**Authors’ Response to Reviewers’ comments and Suggestions**

**Manuscript ID:** PONE-D-21-13888

**Journal:** PLOS ONE

**From**: Authors

**To**: The editor in chief, PLOS ONE

**Version**: II

**Data**: 21/12/2021

**Dear Reviewers**

Thank you so much for giving us an opportunity to submit a revised draft of our manuscript entitled “**Health Status of Street Children and Reasons for Being Forced to Live on the Streets in Harar, Eastern Ethiopia. Using Mixed Methods** to this highly visible impact factor and peer-reviewed Journal. We appreciate the time and effort that you and the reviewers dedicated to providing feedback on our manuscript. We are very grateful for the insightful comments and valuable improvements to our premature paper. We have incorporated most of the **suggestions** and **comments** made by the handling editor and reviewers. All comments and suggestions are clearly stated and well addressed (a point-by-point to the reviewer’s comments and concerns). These changes are **highlighted in Bright Green color** within the clean revised manuscript.

**Authors’ Response to Reviewer 1’s Comments and Suggestions**

**Title: Health Status of Street Children and Reasons for Being Forced to Live on the Streets in Harar, Eastern Ethiopia. Using Mixed Methods**

**To: Reviewer 1**

**From:** Addis Eyeberu (Corresponding Author)

**Subject: Submission of *Incorporated Comments and Suggestions***

First and foremost, we would like to thank you for your constructive and valuable comments and helpful suggestions that helped us to improve and enrich our premature manuscript. Here is the table we have pointed out how authors incorporated your valuable comments, suggestions, and concerns one by one.

| **Reviewer’s Comments to the Authors** | **Authors’ Responses to Reviewer’s comments** |
| --- | --- |
| **Reviewer’s General Comments** | Overall, thank you very much for your positive and constructive suggestions |
| 1. If the authors have adequately addressed your comments raised in a previous round of review and you feel that this manuscript is now acceptable for publication, you may indicate that here to bypass the “Comments to the Author” section, enter your conflict-of-interest statement in the “Confidential to Editor” section, and submit your "Accept" recommendation.  Reviewer #1: (No Response) | **Thank you very much** for your valuable inputs in advancing this manuscript. Now we tried to address all comments and it is incorporated in the revised manuscript. |
| 2. Is the manuscript technically sound, and do the data support the conclusions?  **Reviewer #1: No** | **Thank you very much!!** Now we have tried our best to utilize the comments and suggestions to make the manuscript technically sound and the data support the conclusion. |
| **3.** Has the statistical analysis been performed appropriately and rigorously?  **Reviewer #1: No** | **Thank you so much**. We would like to thank you for your inputs and constructive suggestion. Now, we enriched and revised the paper after initial submission. |
| 4. Have the authors made all data underlying the findings in their manuscript fully available?  **Reviewer #1: Yes** | Thanks! |
| 5. Is the manuscript presented in an intelligible fashion and written in standard English?  **Reviewer #1: No** | **Thank you very much** for such valuable intellectual input. The reviewer is correct. The authors critically considered this input. Now, we thoroughly revised and edited the whole parts of our manuscript and extensively corrected all copy-editing errors in the clean revised manuscript. The authors also sent the manuscript to a language expert/editor who critically reviewed, edited, and corrected all language-related errors made in a submitted manuscript. |
| **6. Specific Review’s Comments to the Authors** | **Authors Responses to Reviewers’ Specific comments** |
| **# Reviewer 1**. What and how this research adds to the literature and help policymaker work for the betterment of street children. | Thank you for your insightful questions about how this research adds to existing literature and what it means for policymakers. This, I believe, is a critical question that must be addressed because it is both the core and the yolk of research's ultimate finding.  This study's findings have a significant and profound impact on the existing literature in one way or another. Here are just a few instances from a long list**.**   - Despite being a pocket study, this study attempted to point out the health status of street children. Because they are vulnerable groups or segments of our population, they have received some attention. As a result, we humbly believe that we are in a position to add this small droplet to the vast ocean of previously acquired knowledge. - We also endeavored to demonstrate why street children are forced to live on the streets. Only studying the health status of our target population is insufficient; as we found them on the street, we need to find out what factors compelled them to leave their home and live on the street. - This study also demonstrates how common divorce was among the parents of street children, which was a previously understudied area. As a result, we attempted to see this and demonstrate how prevalent the problem is.   This research finding could play a critical role in improving the lives of street children for policymakers in some way. to name a few.   - Policymakers must take steps toward reuniting street children with their families. For any child longing to be reunited with his or her family, the emotional and psychological hurdles can be enormous. This is especially true for children who have been reintegrated into their families and communities after spending time on the streets as a result of a variety of issues such as family breakdown, parent death, and poverty. As a result, this study throws at least one stone in the direction of changing the current situation. - By identifying the causes of streetism, policymakers can intervene on the factors that push children to become street children. Poverty, a lack of education, abuse, and a lack of parental care all contribute to the plight of street children. Push-factors such as abuse, domestic violence, or dysfunctional family relationships are common among street children. It may even come to the point where their situation at home becomes unbearable and they choose to live on the street. Therefore, as preventative work reduces the number of children living on the street, organizations must make significant efforts early on. - To curb the phenomenon of street children, policymakers should take steps to socialize this vulnerable segment of our society. This study plays an important role in this regard. - This study assists policymakers in working on the education of street children, allowing those vulnerable segments of the population to shape character, attitude, and behavior that is completely consistent with societal norms and values. - It enables policymakers to devise alternative solutions for street children on how to live their lives in ways other than engaging in criminal activity. - This study appears to assist policymakers in taking action on substance use among street children, which leads to widespread problems for them. |
| 2. You need to report the type of mixed method (e.g., sequential or parallel). | **Thank you very much** for your countless effort to review our premature manuscript. Now we revised the manuscript based on the comment and suggestions. The comment is incorporated in the revised manuscript. |
| **3**.“I want to know the reason for conducting an interview for quantitative research design and for qualitative you have done focus group. | **Dear,** Reviewer, thank you very much for your countless effort. Dear respected reviewer, to be clear from the very outset, we used interviewer-administered questionnaires to collect quantitative data about the participants, while focus group discussions and key informants were used to collect qualitative data. And we have a plenty of reasons for conducting interviewer-administered questionnaires for quantitative research designs, and focus groups for qualitative research designs. We attempted to see and elaborate separately in the sections that follow.  Reason why we undertook Focus group discussion for qualitative research design   - In order to gather information in a short period of time - To uncover participants' perceptions and values about the problem at hand (Health status of street children and reasons forced them to live on the street) - So as to dig out and obtain multiple perspectives on the same topic - To collect data on collective opinions.   Collecting information from carefully chosen groups of people |
| 4. There is no need to mention software in the abstract. | **Thank you so much** for your valuable and insightful comments**.** It is corrected and incorporated in the revised manuscript. |
| 5. You didn’t mention the result heading. For abstract, you can explain results by integrating findings of quantitative and qualitative results. | **Thank you for your comment and question**. We apologize for such kinds of errors. Now it is corrected and incorporated in the revised manuscript. |
| 6. You have explained the problem in the introduction, but you can add literature from a similar context or from the courtiers which have similar culture and context, to explain causes that force street children to stay on the streets. | Thank you for your comment and suggestion. It is corrected and incorporated in the revised manuscript. Now the introduction is revised based on the comments and suggestions. |
| 7. Explain the type of mixed-method design. In the case of phenomenological study, you need to explain the lived experience. What experience you are studying and how. | Thank you for your insightful and wonderful comment. We accept your comment and we incorporate it into the revised manuscript. Since we conduct FGD, their lived experiences were studied and we incorporated the results in the revised manuscript. |
| 8. Sample size determination is fine for quantitative study but for qualitative especially for phenomenological studies, the sample size needs to be selected carefully. | Thank you for your comment. We accept it and corrected it in the revised manuscript. |
| 9. You didn’t mention the name of questionnaires or scales for the quantitative study. Which instrument you adapted. You also have to mention questions of the interview guideline for the focus group. | Thank you for your specific comment. Since we adapted and modified the questionaries from different kinds of literature into the local context, it doesn’t have a specific name however it is a semi-structured questionary. For FGD the sample questions include  **Focus group discussion items**  1. What do you think are the major causes of child Streetism?  2. Can you give details of the difficulties that street children face?  3. can you give details of coping mechanisms of street livelihood?  4. What do you think the multiple agencies should do to help street children?  5. Do you believe the intervention of the agencies dealing with street children and their families is adequate? if not what do you recommend as an improvement |
| 10. **Result:** This section is quite improved; however, I couldn’t understand the integration point for both qualitative and quantitative studies. Especially, if you are reporting that you did a phenomenological study, you need to report lived experiences of the street children. | Thank you for your insightful comment. We revised the result section based on your recommendations. |
| 11. **Discussion:** This study has rich data on the health status of street children that need to be explained and interpreted in detail. There are multiple limitations to the study that you need to report. You need to explain the future recommendation as well. | Thank you for your specific comment. We revised the discussion. Now it is corrected and incorporated in the revised manuscript. |
| End of authors responses for Reviewer 1 | |
| Authors’ Response to Reviewer 2’s Comments and Suggestions | |
| **Reviewer’s General Comments** | Overall, thank you very much for your positive and constructive suggestions |
| 1. If the authors have adequately addressed your comments raised in a previous round of review and you feel that this manuscript is now acceptable for publication, you may indicate that here to bypass the “Comments to the Author” section, enter your conflict-of-interest statement in the “Confidential to Editor” section, and submit your "Accept" recommendation.  **Reviewer #2**: All comments have been addressed | Thank you very much for your valuable inputs in advancing this manuscript. |
| 2. Is the manuscript technically sound, and do the data support the conclusions?  **Reviewer #2: Yes** | **Thank you very much!!** |
| **3.** Has the statistical analysis been performed appropriately and rigorously?  **Reviewer #2: Yes** | **Thank you so much**. We would like to thank you for your appreciation and constructive suggestion. |
| 4. Have the authors made all data underlying the findings in their manuscript fully available?  **Reviewer #2: Yes** | Thanks! |
| 5. Is the manuscript presented in an intelligible fashion and written in standard English?  **Reviewer #2: Yes** | **Thank you very much** for such valuable intellectual input. |

### End of authors responses for Reviewer 2
