# Supplementary material for: Health status of street children and reasons for being forced to live on the streets in Harar, Eastern Ethiopia. Using mixed methods
Source: PLoS One. 2022 Mar 18;17(3):e0265601. doi: 10.1371/journal.pone.0265601 (PMC8932583; doi:10.1371/journal.pone.0265601)
Supplement: Attachment — Submitted filename: Third revision.docx [file pone.0265601.s009.docx]

Authors worked very hard to improve the manuscript, which is depicted in the manuscript. However, I have few comments which authors may incorporate.

1. Please double check language and typing mistakes in the article.
2. Mention the name of the questionnaire you adapted and translated (a (Afan, Oromo, and Amharic)
3. What is the validity and reliability of original version of a (Afan, Oromo, and Amharic)
4. Cross check whether in sequential mixed method, you will have to collect data from the same respondents or not and share the reference of mixed method you have followed.
