# Supplementary material for: Health status of street children and reasons for being forced to live on the streets in Harar, Eastern Ethiopia. Using mixed methods
Source: PLoS One. 2022 Mar 18;17(3):e0265601. doi: 10.1371/journal.pone.0265601 (PMC8932583; doi:10.1371/journal.pone.0265601)
Supplement: Attachment — Submitted filename: Response to reviwers.docx [file pone.0265601.s010.docx]

**Authors’ Response to Reviewers’ comments and Suggestions**

**Manuscript ID:** PONE-D-21-13888

**Journal:** PLOS ONE

**From**: Authors

**To**: The editor in chief, PLOS ONE

**Version**: III

**Date**: 26/2/2022

**Dear Reviewers**

Thank you so much for allowing us to submit a third revised draft of our manuscript entitled “**Health Status of Street Children and Reasons for Being Forced to Live on the Streets in Harar, Eastern Ethiopia. Using Mixed Methods** to this highly visible impact factor and peer-reviewed Journal. We appreciate the time and effort that you and the reviewers dedicated to providing feedback on our manuscript. We are very grateful for the insightful comments and valuable improvements to our premature paper. We have incorporated most of the **suggestions** and **comments** made by the handling editor and reviewers. All comments and suggestions are clearly stated and well addressed (a point-by-point to the reviewer’s comments and concerns). These changes are **highlighted in Bright Green color** within the clean revised manuscript.

**Authors’ Response to Editors and Reviewer 1’s Comments and Suggestions**

**Title: Health Status of Street Children and Reasons for Being Forced to Live on the Streets in Harar, Eastern Ethiopia. Using Mixed Methods**

**To: Editor and Reviewer 1**

**From:** Addis Eyeberu (Corresponding Author)

**Subject: Submission of *Incorporated Comments and Suggestions***

First and foremost, we would like to thank you for your constructive and valuable comments and helpful suggestions that helped us to improve and enrich our premature manuscript. Here is the table we have pointed out how authors incorporated your valuable comments, suggestions, and concerns one by one.

| **Editors comment to Authors** | **Authors’ Responses to Editor’s comments** |
| --- | --- |
| Please review your reference list to ensure that it is complete and correct. If you have cited papers that have been retracted, please include the rationale for doing so in the manuscript text, or remove these references and replace them with relevant current references. Any changes to the reference list should be mentioned in the rebuttal letter that accompanies your revised manuscript. If you need to cite a retracted article, indicate the article’s retracted status in the References list and also include a citation and full reference for the retraction notice. | **Thank you for your comment.** Now the authors reviewed the references list. The reference lists were revised to make them complete and correct based on your recommendation and journal requirement. No article has been retracted. |
| **End of editor’s comment** | |
| **Reviewer’s Comments to the Authors** | **Authors’ Responses to Reviewer’s comments** |
| **Reviewer’s General Comments** | Overall, thank you very much for your positive and constructive suggestions |
| 1. If the authors have adequately addressed your comments raised in a previous round of review and you feel that this manuscript is now acceptable for publication, you may indicate that here to bypass the “Comments to the Author” section, enter your conflict-of-interest statement in the “Confidential to Editor” section, and submit your "Accept" recommendation.  Reviewer #1: All comments have been addressed | **Thank you very much** **again** for your valuable and wonderful input in advancing this manuscript. |
| 2. Is the manuscript technically sound, and do the data support the conclusions?  **Reviewer #1:** Partly | **Thank you very much!!** Now we have tried our best to utilize the comments and suggestions to make the manuscript technically sound and the data support the conclusion. |
| **3.** Has the statistical analysis been performed appropriately and rigorously?  **Reviewer #1: No Response** | **Thank you so much**. We would like to thank you for your input and constructive suggestion. Now, we enriched and revised the paper based on your contrafactive input. |
| 4. Have the authors made all data underlying the findings in their manuscript fully available?  **Reviewer #1: No Response** | **Thanks**! we provide all necessary data to the journal and we made all data underlying the findings in their manuscript fully available. |
| 5. Is the manuscript presented in an intelligible fashion and written in standard English?  **Reviewer #1: Yes** | **Thank you very much** for such valuable intellectual input. |
| **6. Specific Review’s Comments to the Authors** | **Authors' Responses to Reviewers’ Specific comments** |
| **# Reviewer 1**. Please double-check the language and typing mistakes in the article. | Thank you for your insightful comments. Now the authors checked the language and typing errors. The authors critically considered this input. Now, we thoroughly revised and edited the whole parts of our manuscript and extensively corrected all copy-editing errors in the clean revised manuscript. The authors also sent the manuscript to a language expert/editor who critically reviewed, edited, and corrected all language-related errors made in a submitted manuscript. |
| 2. Mention the name of the questionnaire you adapted and translated (Afan Oromo, and Amharic) | **Thank you very much for your countless effort to review our manuscript.** Now we revised the manuscript based on the comments and suggestions. The comments are incorporated in the revised manuscript. We tried to do our best to adapt a tool named “the health status of street children survey” from measure evaluation which was funded by USAID and undertaken with the title of “children in Adverse Situations Indicators and Survey Tools”, available at <https://www.measureevaluation.org/resources/publications/tl-19-35e.html> and from previous studies (BSS tool), which was done by Demelash Habtamu, Addisie Adamu, titled with "Assessment of Sexual and Reproductive Health Status of Street Children in Addis Ababa", *Journal of Sexually Transmitted Diseases*, vol. 2013, Article ID 524076, 20 pages, 2013. <https://doi.org/10.1155/2013/524076> |
| 3. What is the validity and reliability of the original version of (Afan Oromo, and Amharic) | **Dear reviewer, thank you very much for your countless effort. Dear respected reviewer,** we tried to mention the validity and reliability of the original tools (English version) in our first version of comments both on revised documents and response to reviewers. In that time, we considered the whole measure we are expected to do before administering data collection tool to the participants so as to enhance and improve the quality of the study. It is clearly known that, the consistency with which an instrument produces the same results across multiple trials are referred to as its reliability. The degree to which an instrument measures what it was designed to measure is known as its validity. Regarding to the English version of the tool we tried to check the validity and reliability statistically. To this end, we performed Cronbach's Alpha, which is a measure used to assess the quality of our employed instruments. The result was 0.87, which was within acceptable ranges. Nevertheless, we are unable to do statistical test such as Cronbach's Alpha for translated tool since the there is no compatible software that accept both local languages. However, the translated languages were back translated to original version and there was no problem of consistency. |
| 4. Cross check whether in sequential mixed method, you will have to collect data from the same respondents or not and share the reference of mixed-method you have followed. | **Thank you so much for your valuable and insightful comments.**  Dear respected reviewer, here we tried to remained you that during data collection, we followed the steps which was mentioned here in. To this end, we first collected and analyzed the quantitative data and following this we collected and analyzed qualitative data. The purpose was to use qualitative results to assist in explaining and interpreting the findings of a quantitative study. Hence, we used sequential mixed methods which is termed as explanatory sequential mixed methods. This can be done either by re-contacting appropriate members of the original quantitative sample or by drawing a new sample that meets the purposive criteria. The reference we followed was according to **Creswell, 2003 and (Ivankova NV, Creswell JW, Stick SL. Using Mixed-Methods Sequential Explanatory Design: From Theory to Practice. Field Methods. 2006;18(1):3-20).** |
| End of authors responses for Reviewer 1 | |
| Authors’ Response to Reviewer 2’s Comments and Suggestions | |
| **Reviewer’s General Comments** | Overall, thank you very much for your positive and constructive suggestions |
| 1. If the authors have adequately addressed your comments raised in a previous round of review and you feel that this manuscript is now acceptable for publication, you may indicate that here to bypass the “Comments to the Author” section, enter your conflict-of-interest statement in the “Confidential to Editor” section, and submit your "Accept" recommendation.  **Reviewer #2**: All comments have been addressed | Thank you very much for your valuable input in advancing this manuscript. |
| 2. Is the manuscript technically sound, and do the data support the conclusions?  **Reviewer #2: Yes** | **Thank you very much!!** |
| **3.** Has the statistical analysis been performed appropriately and rigorously?  **Reviewer #2: Yes** | **Thank you so much**. We would like to thank you for your appreciation and constructive suggestion. |
| 4. Have the authors made all data underlying the findings in their manuscript fully available?  **Reviewer #2: Yes** | Thanks! |
| 5. Is the manuscript presented in an intelligible fashion and written in standard English?  **Reviewer #2: Yes** | **Thank you very much** for such valuable intellectual input. |

### End of authors responses for Reviewer 2
